# *Legionella*-Containing Vacuoles Capture PtdIns(4)*P*-Rich Vesicles Derived from the Golgi Apparatus

**DOI:** 10.1128/mBio.02420-18

**Published:** 2018-12-11

**Authors:** Stephen Weber, Bernhard Steiner, Amanda Welin, Hubert Hilbi

**Affiliations:** a Institute of Medical Microbiology, University of Zürich, Zürich, Switzerland; Pasteur Institute; Washington State University; Gifu University

**Keywords:** Amoeba, *Dictyostelium*, Golgi apparatus, *Legionella*, effector protein, host-pathogen interaction, live-cell imaging, pathogen vacuole, phosphoinositide lipid, type IV secretion, vesicle trafficking

## Abstract

Legionella pneumophila is the causative agent of a pneumonia termed Legionnaires’ disease. The facultative intracellular bacterium employs the Icm/Dot type IV secretion system (T4SS) and a plethora of translocated “effector” proteins to interfere with host vesicle trafficking pathways and establish a replicative niche, the *Legionella*-containing vacuole (LCV). Internalization of the pathogen and the events immediately ensuing are accompanied by host cell-mediated phosphoinositide (PI) lipid changes and the Icm/Dot-controlled conversion of the LCV from a PtdIns(3)*P*-positive vacuole into a PtdIns(4)*P*-positive replication-permissive compartment, which tightly associates with the endoplasmic reticulum. The source and formation of PtdIns(4)*P* are ill-defined. Using dually labeled Dictyostelium discoideum amoebae and real-time high-resolution confocal laser scanning microscopy (CLSM), we show here that nascent LCVs continuously capture and accumulate PtdIns(4)*P*-positive vesicles from the host cell. Trafficking of these PtdIns(4)*P*-positive vesicles to LCVs occurs independently of the Icm/Dot system, but their sustained association requires a functional T4SS. During the infection, PtdIns(3)*P*-positive membranes become compacted and segregated from the LCV, and PtdIns(3)*P*-positive vesicles traffic to the LCV but do not fuse. Moreover, using eukaryotic and prokaryotic PtdIns(4)*P* probes (2×PH_FAPP_-green fluorescent protein [2×PH_FAPP_-GFP] and P4C_SidC_-GFP, respectively) along with Arf1-GFP, we show that PtdIns(4)*P*-rich membranes of the *trans*-Golgi network associate with the LCV. Intriguingly, the interaction dynamics of 2×PH_FAPP_-GFP and P4C_SidC_-GFP are spatially separable and reveal the specific PtdIns(4)*P* pool from which the LCV PI originates. These findings provide high-resolution real-time insights into how L. pneumophila exploits the cellular dynamics of membrane-bound PtdIns(4)*P* for LCV formation.

## Abstract (precis)

The environmental bacterium Legionella pneumophila causes a life-threatening pneumonia termed Legionnaires’ disease. The bacteria grow intracellularly in free-living amoebae as well as in respiratory tract macrophages. To this end, L. pneumophila forms a distinct membrane-bound compartment called the *Legionella*-containing vacuole (LCV). Phosphoinositide (PI) lipids are crucial regulators of the identity and dynamics of host cell organelles. The PI lipid PtdIns(4)*P* is a hallmark of the host cell secretory pathway, and decoration of LCVs with this PI is required for pathogen vacuole maturation. The source, dynamics, and mode of accumulation of PtdIns(4)*P* on LCVs are largely unknown. Using *Dictyostelium* amoebae producing different fluorescent probes as host cells, we show here that LCVs rapidly acquire PtdIns(4)*P* through the continuous interaction with PtdIns(4)*P*-positive host vesicles derived from the Golgi apparatus. Thus, the PI lipid pattern of the secretory pathway contributes to the formation of the replication-permissive pathogen compartment.

## INTRODUCTION

The causative agent of a life-threatening pneumonia called Legionnaires’ disease, Legionella pneumophila, is a natural parasite of environmental protozoa, including *Acanthamoeba* and *Dictyostelium* spp. (1–4). L. pneumophila is a facultative intracellular pathogen, which in amoebae as well as in mammalian macrophages replicates in a dedicated compartment, the *Legionella*-containing vacuole (LCV) (5–7). LCV formation is a complex process depending on the bacterial Icm/Dot type IV secretion system (T4SS) (8), which translocates a plethora of T4SS substrates termed “effector” proteins into host cells, where they subvert critical processes (9–11).

The LCV avoids fusion with bactericidal lysosomes but extensively communicates with the endocytic, secretory, and retrograde trafficking pathways and eventually is tightly engulfed by the endoplasmic reticulum (ER) (6, 12, 13). Small GTPases of the Arf (14, 15), Rab (6, 16), Ran (17), and Rap (18) families regulate organelle and cell dynamics and play important roles for L. pneumophila-host cell interactions. Furthermore, large GTPases implicated in membrane fusion and fission events contribute to L. pneumophila infection. The ER tubule-resident large GTPase atlastin3 (Atl3/Sey1) promotes ER remodeling around LCVs, pathogen vacuole expansion, and intracellular replication (19), and the large dynamin1-like GTPase Dnm1l mediates L. pneumophila-induced mitochondrial fragmentation and inhibition of respiration (20).

Another crucial class of regulators of membrane dynamics comprises the phosphoinositide (PI) lipids. These mono- or polyphosphorylated derivatives of phosphatidylinositol (PtdIns) are present in low abundance in all cell membranes and codetermine organelle identity and vesicle trafficking routes (21, 22). The turnover of PI lipids is tightly controlled in a spatiotemporal manner by PI kinases and phosphatases. Seven naturally occurring PI lipids exist, among which PtdIns(4,5)*P*_2_ governs the connection of the cytoskeleton to the plasma membrane, and PtdIns(3)*P* or PtdIns(4)*P* represent pivotal regulators of the endocytic or secretory (anterograde) trafficking pathway, respectively. PtdIns(4)*P* is the key PI lipid component of the Golgi apparatus (23) but is also present at the plasma membrane and (late) endosomes (22, 24).

Live-cell imaging of the spatiotemporal PI pattern in L. pneumophila-infected D. discoideum revealed that a PtdIns(3,4,5)*P*_3_-rich cup is formed during uptake, immediately followed by the formation of a PtdIns(3,4,5)*P*_3_-rich macropinosome (25). Regardless of whether the compartment contains virulent L. pneumophila or an Icm/Dot mutant strain, PtdIns(3,4,5)*P*_3_ disappears within a minute, and PtdIns(4,5)*P*_2_ is regenerated at the site of uptake. Up to 30 min after uptake, LCVs harboring virulent or Icm/Dot mutants accumulate PtdIns(3)*P*, the volume of the macropinosome lumen concomitantly decreases, and the LCV appears tight. While LCVs harboring Icm/Dot mutants remain PtdIns(3)*P*-positive, LCVs harboring wild-type L. pneumophila gradually lose PtdIns(3)*P*, which still decorates about 20% of the vacuoles at 2 h postinfection (p.i.). Beyond 2 h, the LCV continues to expand, and PtdIns(3)*P* becomes undetectable. Remarkably, LCVs steadily acquire PtdIns(4)*P*, and the PI remains on the pathogen vacuole membrane throughout the infection (25, 26). At 2 h p.i., nearly all LCVs are positive for PtdIns(4)*P* and appear spherical with a very intense ring of this PI. Of note, the LCVs acquire PtdIns(4)*P* prior to and independently of the ER, and the two membranes remain distinct over a long period of time during the infection (25). Except for a weak and transient localization of plasma membrane-derived PtdIns(4)*P*, this PI is not present on the tight vacuoles harboring Icm/Dot mutant L. pneumophila.

The PI conversion from PtdIns(3)*P* to PtdIns(4)*P* is a hallmark of LCV maturation (19, 25–27). PtdIns(4)*P* is bound by a number of L. pneumophila effectors, which, due to different catalytic activities and host targets, further promote the maturation of the pathogen vacuole. PtdIns(4)*P*-binding Icm/Dot substrates include SidC (26, 28, 29) and SidM (alias DrrA) (30–34). However, the source, dynamics, and mode of accumulation of PtdIns(4)*P* on LCVs are ill-defined. To address this issue, we used high-resolution live-cell imaging of L. pneumophila-infected, dually labeled D. discoideum amoebae. Here, we reveal that nascent LCVs continuously capture and accumulate PtdIns(4)*P*-positive, Golgi-derived vesicles from the host cell. While the interaction of pathogen vacuoles with PtdIns(4)*P*-positive vesicles occurs independently of the bacterial Icm/Dot T4SS, the sustained association of the vesicles with LCVs requires a functional T4SS.

## RESULTS

### Early LCVs capture host-derived PtdIns(4)*P*-rich vesicles.

**FIG 1:**
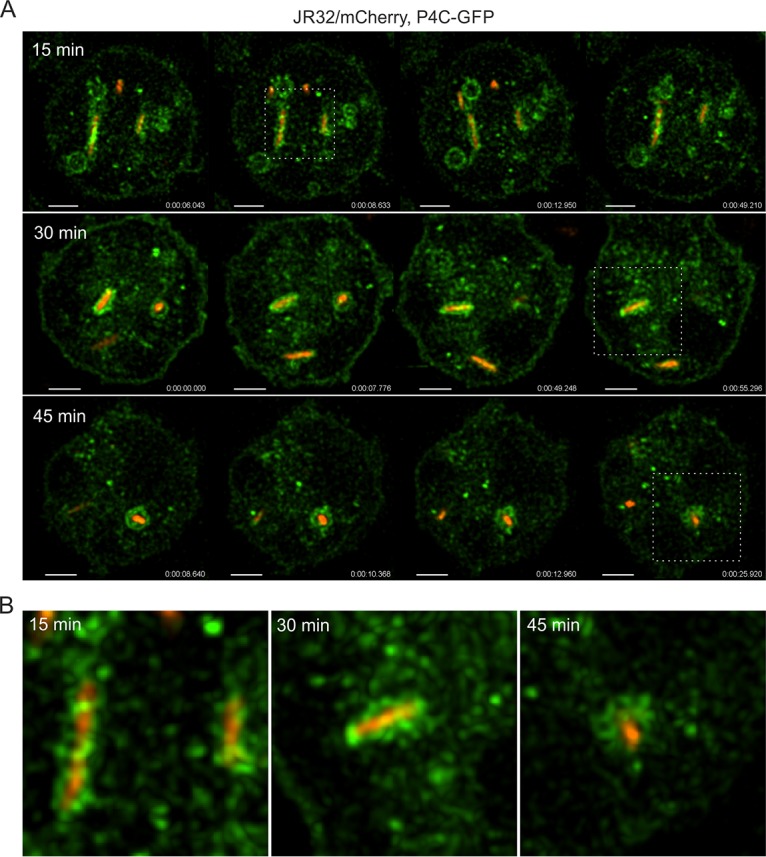
Early LCVs capture host-derived PtdIns(4)*P*-rich vesicles. (A) D. discoideum Ax3 amoebae producing P4C_SidC_-GFP (pWS034) were infected (MOI 5) with L. pneumophila JR32 producing mCherry (pNP102). Frames were taken from three-dimensional resonant CLSM videos at 15 min (see Movie S1), 30 min (Movie S2), and 45 min (movie not shown) postinfection (p.i.). (B) Expanded magnified views correspond to the areas indicated by the white boxes in panel A. Time scale, hours:minutes:seconds:milliseconds (h:m:s:ms). Scale bars, 2 µm.

The secretory pathway PI lipid, PtdIns(4)*P*, was previously shown to visibly accumulate on the LCV around 30 min p.i. (25). However, it is not known whether the PI lipid is formed directly on the LCV by phosphorylation or dephosphorylation of a PI precursor molecule or whether it accumulates on the LCV by interaction with PtdIns(4)*P*-rich membranes. To address this issue, we used D. discoideum amoebae producing P4C_SidC_-GFP, a PtdIns(4)*P*-specific probe comprising the PI-binding domain of the L. pneumophila Icm/Dot substrate SidC (28, 35). The amoebae were infected with red fluorescent L. pneumophila and analyzed by real-time three-dimensional (3D) resonant confocal laser scanning microscopy (CLSM). A fast capture rate of 5 frames per second revealed the speed and dynamics of PtdIns(4)*P* trafficking to the LCV, and 3D capture over time allowed the visualization of compartment lumen above and below the standard plane of focus in 2D (Fig. 1; see also Movies S1 and S2).

10.1128/mBio.02420-18.1 MOVIE S1 Early LCVs capture host-derived PtdIns(4)*P*-rich vesicles; CLSM video of D. discoideum Ax3 amoebae producing P4C_SidC_-GFP infected for 15 min with L. pneumophila JR32 producing mCherry. Download Movie S1, MOV file, 2.7 MB. Copyright © 2018 Weber et al. 2018 Weber et al. This content is distributed under the terms of the Creative Commons Attribution 4.0 International license.

10.1128/mBio.02420-18.2 MOVIE S2 Early LCVs capture host-derived PtdIns(4)*P*-rich vesicles; CLSM video of D. discoideum Ax3 amoebae producing P4C_SidC_-GFP infected for 30 min with L. pneumophila JR32 producing mCherry. Download Movie S2, MOV file, 1.8 MB. Copyright © 2018 Weber et al. 2018 Weber et al. This content is distributed under the terms of the Creative Commons Attribution 4.0 International license.

The high temporal and spatial resolution of the 3D-CLSM approach demonstrated the dynamic and transient association of PtdIns(4)*P*-positive vesicles with the LCV, ultimately resulting in a net accumulation of vesicles. Using this approach, at 15 min p.i., PtdIns(4)*P* accumulation at the LCV was already evident (Fig. 1A; see also Movie S1 in the supplemental material). The PtdIns(4)*P* signal showed a heterogeneous distribution, and the PtdIns(4)*P*-rich vesicles did not assume any fixed position. The image insets demonstrate the vesicular nature of the PtdIns(4)*P* association (Fig. 1B). At 30 min p.i., net accumulation of PtdIns(4)*P*-rich vesicles was obvious, increasingly giving the appearance that the PtdIns(4)*P* around the LCV was a continuous membrane (Fig. 1; see also Movie S2). However, this was not the case; individual vesicles could still be resolved, and the vesicle association did not show a continuous elliptical curvature, as typically observed with longer exposure times.

By 45 min p.i., the LCV took on the classic spherical appearance. The LCV membrane comprised a collection of slightly larger PtdIns(4)*P*-positive vesicles, compared to the previous time points (Fig. 1) (movie not shown). Importantly, the 45-min time series clearly illustrates that the PtdIns(4)*P* association is vesicular, as the vesicles could be observed to change position and deviate from the limiting LCV membrane, rather than forming a continuous PtdIns(4)*P*-positive membrane. The image inset of the final frame poignantly confirms these observations, as the individual PtdIns(4)*P* vesicle lumens became resolvable in their dynamic repositioning. In summary, the use of real-time 3D high-resolution resonant CLSM allowed the observation of the net accumulation of PtdIns(4)*P*-rich vesicles on LCVs. At around 45 min, vesicle lumens were still resolvable, and LCVs were not uniformly coated with a continuous PtdIns(4)*P* membrane. Rather, vesicles “stagnated” on most LCVs, thus apparently leading to a net accumulation of the PtdIns(4)*P* lipid.

### Host- and T4SS-dependent association of PtdIns(4)*P* vesicles with LCVs.

**FIG 2:**
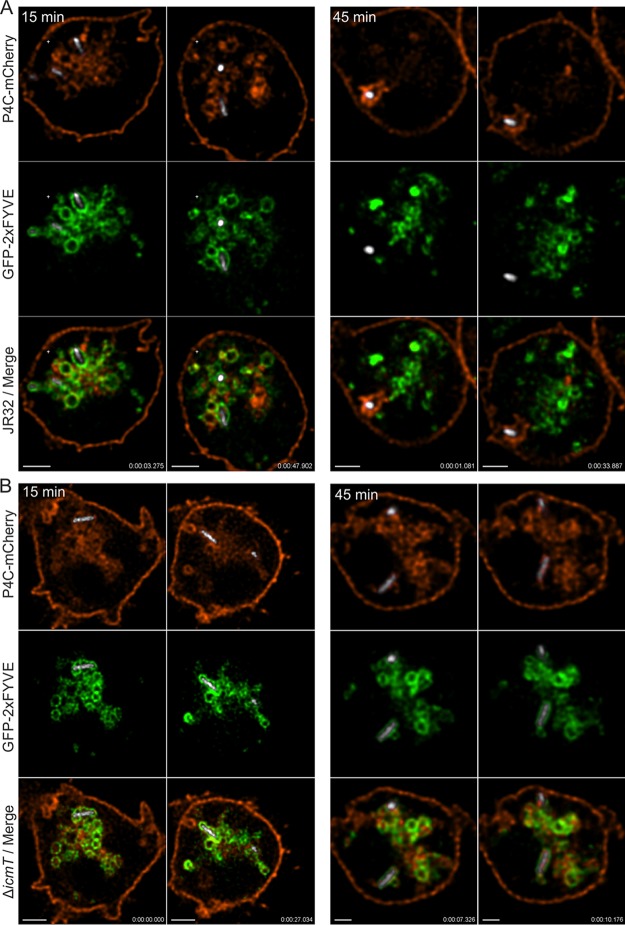
Host- and T4SS-dependent association of PtdIns(4)*P* vesicles with LCVs. D. discoideum Ax3 amoebae producing GFP-2×FYVE (pHK95) and P4C_SidC_-mCherry (pWS032) were infected (MOI 5) with (A) L. pneumophila JR32 (Movie S3 and S4) or (B) Δ*icmT* (Movie S5 and S6) producing mCerulean (pNP099). Resonant CLSM videos were taken at 15 min (Movie S3 and S5) or 45 min p.i. (Movie S4 and S6). Time scale, h:m:s:ms. Scale bars, 2 µm.

Applying real-time CLSM, we used dually labeled D. discoideum strains producing in tandem P4C_SidC_-mCherry and the PtdIns(3)*P* probe GFP-2×FYVE to analyze the PI patterns underlying the formation of vacuoles harboring L. pneumophila JR32 or the T4SS-deficient strain Δ*icmT*. The high-resolution approach revealed that vesicles positive for PtdIns(4)*P* or PtdIns(3)*P* both simultaneously and independently of one another interacted with the bacterial compartments, while the morphological appearances of the vesicles were similar (Fig. 2; see also Movies S3 to S6).

10.1128/mBio.02420-18.3 MOVIE S3 Host- and T4SS-dependent association of PtdIns(4)*P* vesicles with LCVs; CLSM video of D. discoideum Ax3 amoebae producing GFP-2×FYVE and P4C_SidC_-mCherry infected for 15 min with L. pneumophila JR32 producing mCerulean. Download Movie S3, MOV file, 6.6 MB. Copyright © 2018 Weber et al. 2018 Weber et al. This content is distributed under the terms of the Creative Commons Attribution 4.0 International license.

10.1128/mBio.02420-18.4 MOVIE S4 Host- and T4SS-dependent association of PtdIns(4)*P* vesicles with LCVs; CLSM video of D. discoideum Ax3 amoebae producing GFP-2×FYVE and P4C_SidC_-mCherry infected for 45 min with L. pneumophila JR32 producing mCerulean. Download Movie S4, MOV file, 3.2 MB. Copyright © 2018 Weber et al. 2018 Weber et al. This content is distributed under the terms of the Creative Commons Attribution 4.0 International license.

10.1128/mBio.02420-18.5 MOVIE S5 Host- and T4SS-dependent association of PtdIns(4)*P* vesicles with LCVs; CLSM video of D. discoideum Ax3 amoebae producing GFP-2×FYVE and P4C_SidC_-mCherry infected for 15 min with L. pneumophila Δ*icmT* producing mCerulean. Download Movie S5, MOV file, 3.5 MB. Copyright © 2018 Weber et al. 2018 Weber et al. This content is distributed under the terms of the Creative Commons Attribution 4.0 International license.

10.1128/mBio.02420-18.6 MOVIE S6 Host- and T4SS-dependent association of PtdIns(4)*P* vesicles with LCVs; CLSM video of D. discoideum Ax3 amoebae producing GFP-2×FYVE and P4C_SidC_-mCherry infected for 45 min with L. pneumophila Δ*icmT* producing mCerulean. Download Movie S6, MOV file, 1.5 MB. Copyright © 2018 Weber et al. 2018 Weber et al. This content is distributed under the terms of the Creative Commons Attribution 4.0 International license.

At 15 min p.i., PtdIns(3)*P* vesicles associated with early LCVs harboring L. pneumophila JR32, which were not extensively overlapping with the PtdIns(4)*P* vesicles (Fig. 2A; see also Movie S3). Overall, the PtdIns(3)*P* vesicles seemed to associate less tightly with the pathogen compartment than the PtdIns(4)*P* vesicles (Movie S3). Moreover, the net clearance of PtdIns(3)*P* appeared to take place through the shedding of PtdIns(3)*P*-rich vesicles. At 45 min p.i., the PtdIns(3)*P*-positive vesicles were compacted and remained clear of the LCV after their shedding (Movie S4). In contrast, a strong PtdIns(4)*P* signal was observed around the LCV at this time point, in agreement with the concept of dynamic stagnation and net accumulation of PtdIns(4)*P*-rich vesicles. Since the clearance of PtdIns(3)*P* vesicles coincided with the accumulation of PtdIns(4)*P* vesicles, our results indicate that the PI conversion from PtdIns(3)*P* to PtdIns(4)*P* on LCVs takes place through selective vesicle trafficking events rather than as a result of (or in addition to) a direct transformation of PtdIns(3)*P* into PtdIns(4)*P*.

In contrast to LCVs harboring the virulent JR32 strain, vacuoles containing Icm/Dot-deficient Δ*icmT* mutant bacteria remained enriched for PtdIns(3)*P* and, at 15 min p.i. as well as at 45 min p.i., seemed to still acquire PtdIns(3)*P*-positive vesicles (Fig. 2B; see also Movie S5 and S6). Interestingly, PtdIns(4)*P*-positive vesicles also temporarily associated with Δ*icmT*-containing vacuoles, in an obviously Icm/Dot-independent manner, but did not accumulate. At both 15 and 45 min p.i., vesicular PtdIns(4)*P* trafficking to the bacterial compartment was evident, and the early bacterial vacuole was literally dragged through a PtdIns(4)*P*-rich vesicle network. However, in contrast to vacuoles harboring strain JR32, Δ*icmT*-containing vacuoles remained essentially free of immobilized PtdIns(4)*P* (Fig. 2B; see also Movie S5 and S6). Hence, real-time microscopy revealed the fast kinetics of in-coming and out-going vesicle trafficking on LCVs at unprecedented resolution. From these observations, we conclude that there is Icm/Dot-independent “baseline” trafficking of PtdIns(4)*P* vesicles to vacuoles harboring newly internalized bacteria but the Icm/Dot T4SS is necessary for capturing and incorporating these vesicles, thus altering and defining the vacuole identity. Taking the results together, vesicular trafficking largely contributes to both the Icm/Dot-dependent removal and segregation of PtdIns(3)*P* as well as the accumulation of PtdIns(4)*P* on LCVs.

### PtdIns(3)*P*-positive vesicles interact with but do not fuse with PtdIns(4)*P*-positive LCVs.

**FIG 3:**
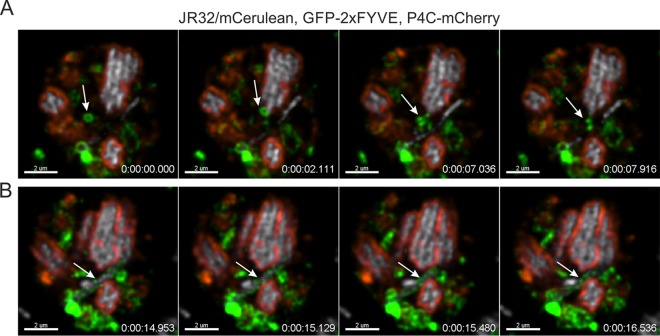
PtdIns(3)*P*-positive vesicles interact with but do not fuse with PtdIns(4)*P*-positive LCVs. D. discoideum Ax3 amoebae producing GFP-2×FYVE (pHK95) and P4C_SidC_-mCherry (pWS032) were infected (MOI 5, 18 h) with L. pneumophila JR32 producing mCerulean (pNP099). Arrows indicate (A) vesicle trafficking events or (B) sustained PtdIns(3)*P* vesicle association with a PtdIns(4)*P*-negative LCV. Resonant CLSM video was taken at 18 h p.i. (Movie S7). Time scale, h:m:s:ms. Scale bars, 2 µm.

LCVs harboring wild-type L. pneumophila shed their PtdIns(3)*P* identity early during the infection process through the net loss of PtdIns(3)*P*-positive vesicles (Fig. 2). To assess vesicle dynamics at later stages of LCV maturation, we infected dually labeled D. discoideum strains producing P4C_SidC_-mCherry and GFP-2×FYVE in tandem with the virulent JR32 strain and imaged the infection after 18 h. At that time point, all observed LCVs harboring several bacteria were exclusively PtdIns(4)*P*-positive. Under those conditions, PtdIns(3)*P*-rich vesicles still trafficked to PtdIns(4)*P*-positive LCVs but did not fuse or accumulate on the LCV membrane at all (Fig. 3A). Moreover, in heavily infected amoebae, PtdIns(3)*P*-positive vesicles also interacted with PtdIns(4)*P*-negative (likely newly formed) pathogen vacuoles but also did not fuse with these compartments (Fig. 3B).

10.1128/mBio.02420-18.7 MOVIE S7 PtdIns(3)*P*-positive vesicles interact with but do not fuse with PtdIns(4)*P*-positive LCVs; CLSM video of D. discoideum Ax3 amoebae producing GFP-2×FYVE and P4C_SidC_-mCherry infected for 18 h with L. pneumophila JR32 producing mCerulean. Download Movie S7, MOV file, 2.4 MB. Copyright © 2018 Weber et al. 2018 Weber et al. This content is distributed under the terms of the Creative Commons Attribution 4.0 International license.

In general, at a given late point during infection, PtdIns(3)*P*-positive vesicles were still vividly trafficking along microtubules and overall vesicle trafficking seemed intact (Fig. 3; see also Movie S7). These observations indicated that the infection with L. pneumophila was relatively stealthy and did not severely compromise crucial cellular trafficking pathways. In contrast, the trafficking of PtdIns(4)*P*-rich vesicles was no longer observed at late stages of infection, likely because the probe was tied up on the massively PtdIns(4)*P*-positive LCVs at this time point. In summary, at late stages of infection, PtdIns(3)*P*-positive vesicles still interact with but do not fuse with PtdIns(4)*P*-positive LCVs, and the trafficking of these vesicles as well as vesicle trafficking in general does not seem to be substantially compromised by the infection with L. pneumophila.

### LCVs interact with PtdIns(4)*P* from the *trans*-Golgi network.

**FIG 4:**
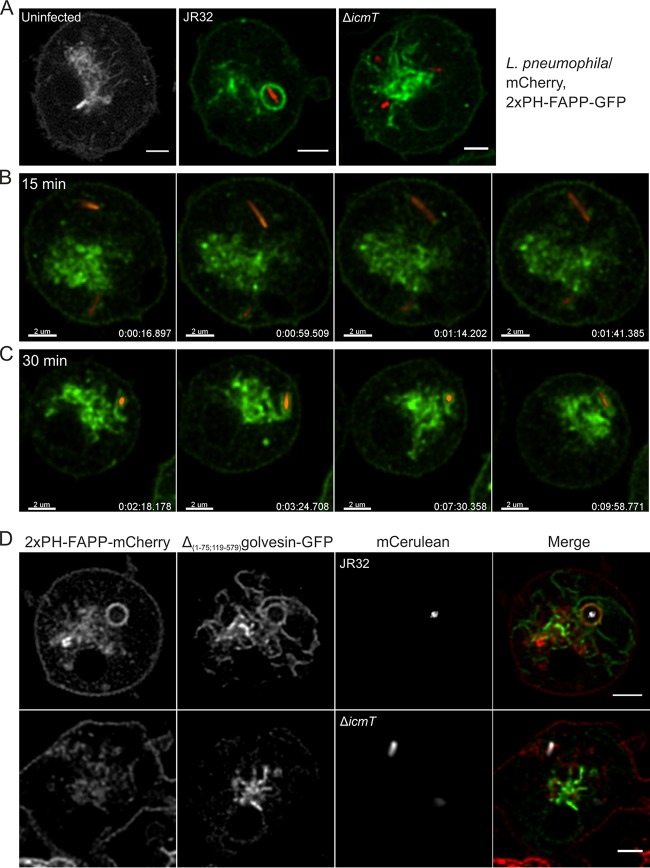
LCVs interact with PtdIns(4)*P* from the *trans*-Golgi network. (A) D. discoideum Ax3 amoebae producing 2×PH_FAPP_-GFP (pWS033) were infected (MOI 5, 2 h) with mCherry-producing L. pneumophila JR32 or Δ*icmT* (pNP102) or left uninfected, and localization of the probe to the Golgi pool of PtdIns(4)*P* and LCVs was observed by CLSM. (B and C) Filaments labeled by 2×PH_FAPP_-GFP in D. discoideum (B) began to associate with the LCV around 15 min p.i. and (C) robustly enveloped the LCV 30 min p.i. (D) D. discoideum Ax3 amoebae producing 2×PH_FAPP_-mCherry (pWS035) and the specific Golgi core probe Δ(1–75;119–579)golvesin-GFP (pWS037) were infected (MOI 5, 1 h) with L. pneumophila JR32 or Δ*icmT* producing mCerulean (pNP099). Scale bars, 2 µm.

To characterize the cellular compartment source of the PtdIns(4)*P*-positive vesicles accumulating on LCVs, we employed D. discoideum strains producing the well-characterized PtdIns(4)*P*/Golgi probe 2×PH_FAPP_-GFP (24, 36). In keeping with the reported probe localization in mammalian cells, 2×PH_FAPP_-GFP principally localizes to the *trans*-Golgi network (TGN), with weak plasma membrane localization also in D. discoideum (Fig. 4A). Upon infection of D. discoideum producing 2×PH_FAPP_-GFP with L. pneumophila JR32, the 2×PH_FAPP_-GFP probe not only labeled the PtdIns(4)*P*-positive filaments of the Golgi apparatus but also accumulated on the limiting membrane of LCVs. Projections of the Golgi apparatus labeled by 2×PH_FAPP_-GFP made contact with and began to associate with the LCV around 15 min p.i. (Fig. 4B) and robustly enveloped the pathogen vacuole 30 min p.i. over the course of several minutes (Fig. 4C). In contrast, in D. discoideum infected with Δ*icmT* mutant bacteria, the probe still robustly labeled the PtdIns(4)*P*-positive filaments of the Golgi apparatus but did not localize to or accumulate on the membrane of vacuoles harboring the avirulent bacteria (Fig. 4A).

To validate the observed interactions of LCVs with Golgi membranes, we used an unrelated Golgi marker, golvesin (37). D. discoideum amoebae producing in parallel 2×PH_FAPP_-mCherry and the specific Golgi core probe Δ(1–75;119–579)golvesin-GFP were infected with L. pneumophila JR32 or Δ*icmT* mutant bacteria for 1 h (Fig. 4D). Vacuoles harboring strain JR32 robustly stained positive for this set of Golgi markers, corroborating that the PtdIns(4)*P* decorating LCVs originated from a Golgi-derived source. In contrast, vacuoles containing Δ*icmT* mutant bacteria were totally devoid of either of the two Golgi markers. Taking the results together, the mammalian PtdIns(4)*P* probe 2×PH_FAPP_-GFP also labels Golgi PtdIns(4)*P* and LCVs in D. discoideum, and the D. discoideum Golgi marker golvesin accumulates on LCVs, indicating that PtdIns(4)*P*-rich Golgi membranes associate with LCVs.

### The Icm/Dot T4SS determines sustained association of LCVs with the Golgi apparatus.

Next, we sought to assess the contribution of the Icm/Dot T4SS to the accumulation of Golgi-derived PtdIns(4)*P*-positive vesicles on LCVs. To this end, we employed D. discoideum strains producing in tandem 2×PH_FAPP_-mCherry and Arf1-GFP. The Golgi-associated small GTPase Arf1 regulates Golgi-ER trafficking as well as *intra*-Golgi transport (38) and is recruited to LCVs by the Icm/Dot translocated effector protein RalF (14).

**FIG 5:**
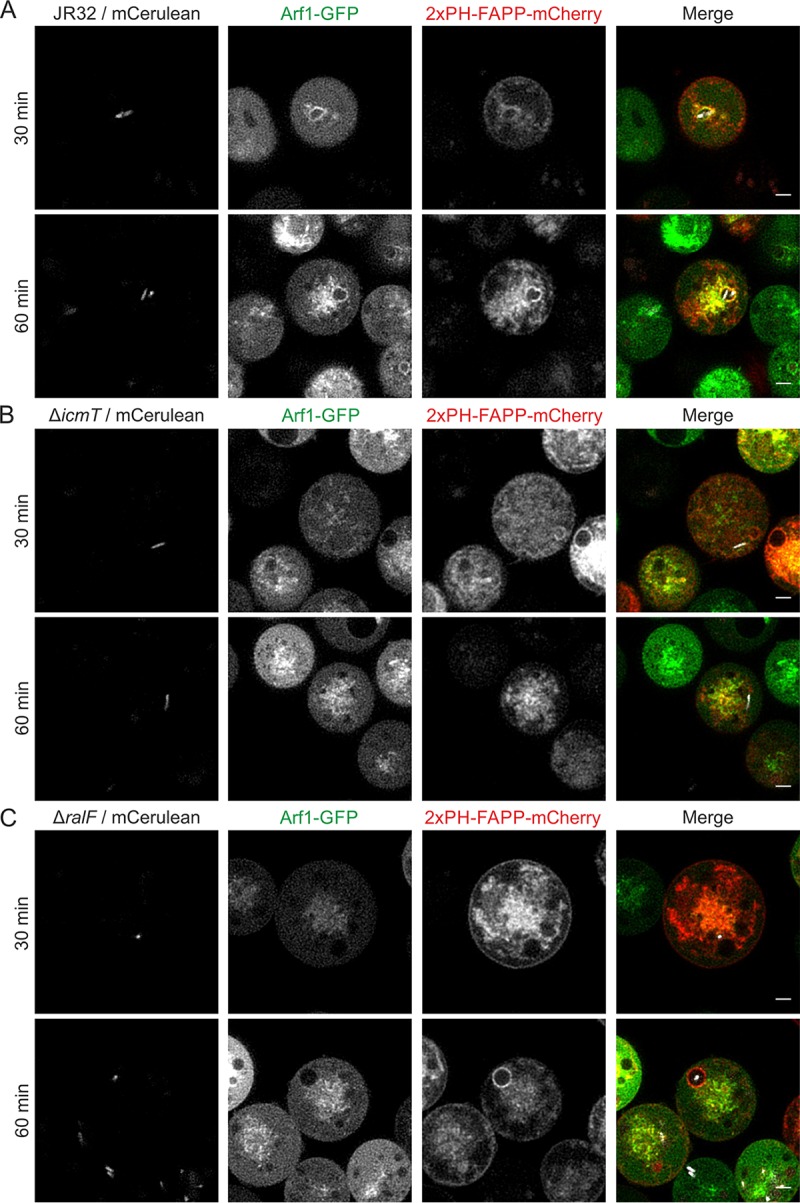
The Icm/Dot T4SS determines sustained association of LCVs with the Golgi apparatus. D. discoideum Ax3 amoebae producing Arf1-GFP (pWS036) and 2×PH_FAPP_-mCherry (pWS035) were infected (MOI 5) with L. pneumophila (A) JR32 (Movie S8), (B) Δ*icmT* (Movie S9), or (C) Δ*ralF* producing mCerulean (pNP099). Sustained and/or transient interactions of both probes simultaneously with bacterium-containing vacuoles were recorded by resonant CLSM at 30 min (movie not shown) and 60 min (Movie S8 and S9) p.i. Time scale, h:m:s:ms. Scale bars, 2 µm.

Upon infection of the dually labeled D. discoideum strain with L. pneumophila JR32, both 2×PH_FAPP_-mCherry and Arf1-GFP associated with LCVs in a sustained manner, but the two probes did not strictly overlap and showed distinct accumulation kinetics (Fig. 5A; see also Movie S8). While the amount of 2×PH_FAPP_-mCherry increased from 30 to 60 min p.i., Arf1-GFP association did not appear to intensify during this period. In contrast, upon infection of D. discoideum producing 2×PH_FAPP_-mCherry and Arf1-GFP with L. pneumophila Δ*icmT*, the Golgi membranes were inevitably brought into proximity of the compartment harboring the bacteria but did not engage in sustained interactions (Fig. 5B; see also Movie S9). The video frames 30 min p.i. showed what appears to be co-localization of the Δ*icmT*-containing compartment and both Golgi probes, but approximately 700 ms later, the Golgi membranes were entirely clear of the compartment. Thus, the Golgi does not sustainably associate with the vacuole containing avirulent L. pneumophila. Finally, upon infection of D. discoideum producing 2×PH_FAPP_-mCherry and Arf1-GFP with L. pneumophila Δ*ralF*, the PtdIns(4)*P* probe labeled LCVs harboring the mutant strain to the same extent as LCVs harboring the parental strain, while Arf1-GFP was not observable on pathogen vacuoles (Fig. 5C). These findings are in agreement with the notion that Golgi-derived PtdIns(4)*P* accumulates on LCVs independently of RalF-mediated Arf1 recruitment. In summary, the use of 2×PH_FAPP_-mCherry and Arf1-GFP revealed that the Icm/Dot T4SS determines sustained association of LCVs with the Golgi apparatus in an Arf1-independent manner.

10.1128/mBio.02420-18.8 MOVIE S8 The Icm/Dot T4SS determines sustained association of LCVs with the Golgi apparatus; CLSM video of D. discoideum Ax3 amoebae producing Arf1-GFP and 2×PH-FAPP-mCherry infected for 60 min with L. pneumophila JR32 producing mCerulean. Download Movie S8, MOV file, 3.3 MB. Copyright © 2018 Weber et al. 2018 Weber et al. This content is distributed under the terms of the Creative Commons Attribution 4.0 International license.

10.1128/mBio.02420-18.9 MOVIE S9 The Icm/Dot T4SS determines sustained association of LCVs with the Golgi apparatus; CLSM video of D. discoideum Ax3 amoebae producing Arf1-GFP and 2×PH-FAPP-mCherry infected for 60 min with L. pneumophila Δ*icmT* producing mCerulean. Download Movie S9, MOV file, 4.7 MB. Copyright © 2018 Weber et al. 2018 Weber et al. This content is distributed under the terms of the Creative Commons Attribution 4.0 International license.

### The PtdIns(4)*P* probes, 2×PH_FAPP_ and P4C_SidC_ show distinct LCV interaction dynamics.

**FIG 6:**
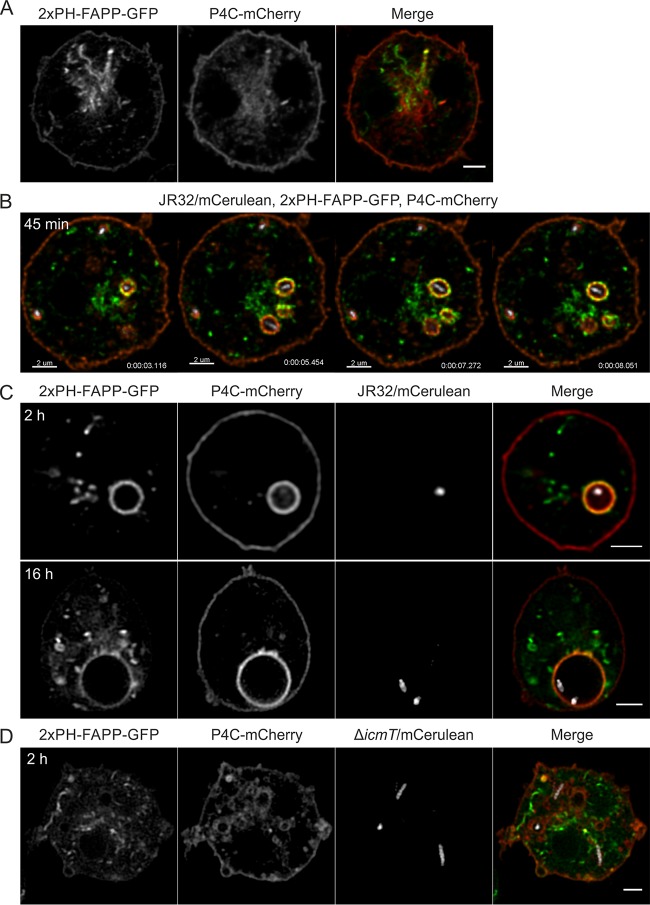
The PtdIns(4)*P* probes, 2×PH_FAPP_ and P4C_SidC_, show distinct LCV interaction dynamics. D. discoideum Ax3 amoebae producing 2×PH_FAPP_-GFP (pWS033) and P4C_SidC_-mCherry (pWS032) were (A) left uninfected or infected (MOI 5) for the time indicated with (B and C) L. pneumophila JR32 or with (D) Δ*icmT* mutant bacteria producing mCerulean (pNP099). PH_FAPP_ predominantly labels the TGN, while P4C_SidC_ predominantly localizes to the plasma membrane and to cytoplasmic vesicles surrounding the Golgi, as well as to LCVs. High-resolution video capture of resonant CLSM is shown (B; Movie S10). Time scale, h:m:s:ms. Scale bars, 2 µm.

Based on the different spatiotemporal localization of 2×PH_FAPP_-mCherry and Arf1-GFP on LCVs, we decided to simultaneously assess the localization dynamics of the eukaryotic and bacterial PtdIns(4)*P* probes, 2×PH_FAPP_ and P4C_SidC_, respectively. In D. discoideum producing in parallel 2×PH_FAPP_-GFP and P4C_SidC_-mCherry, the former predominantly labels the Golgi apparatus, while the latter in addition to the Golgi primarily localizes to the plasma membrane and (endosomal) vesicles surrounding the Golgi (Fig. 6A). Hence, aside from the plasma membrane where P4C_SidC_-mCherry localization is dominant, there is little obvious spatial overlap between the two probes recognizing the same PI lipid.

10.1128/mBio.02420-18.10 MOVIE S10 The PtdIns(4)*P* probes, 2×PH_FAPP_ and P4C_SidC_, show distinct LCV interaction dynamics; CLSM video of D. discoideum Ax3 amoebae producing 2×PH_FAPP_-GFP and P4C_SidC_-mCherry infected for 45 min with L. pneumophila JR32 producing mCerulean. Download Movie S10, MOV file, 0.9 MB. Copyright © 2018 Weber et al. 2018 Weber et al. This content is distributed under the terms of the Creative Commons Attribution 4.0 International license.

Upon infection of D. discoideum producing 2×PH_FAPP_-GFP and P4C_SidC_-mCherry with L. pneumophila JR32, the LCVs were marked by PtdIns(4)*P*-positive vesicles as indicated by P4C_SidC_-mCherry, but were also entangled by a dynamic meshwork of TGN labeled by 2×PH_FAPP_-GFP (Fig. 6B; see also Movie S10). Noteworthy, while P4C_SidC_ exclusively labeled the limiting LCV membrane, thus defining its identity, 2×PH_FAPP_ not only labeled the LCV membrane (as seen in Fig. 4 and 5), but also extended into the TGN. The kinetics of LCV labeling of both probes, P4C_SidC_-mCherry and 2×PH_FAPP_-GFP, were very similar (80% to 90% positive LCVs 1 to 2 h p.i.), and the probes maintained their distinct labeling patterns throughout the infection with L. pneumophila from 2 h p.i. to 16 h p.i. (Fig. 6C).

Upon infection of D. discoideum producing 2×PH_FAPP_-GFP and P4C_SidC_-mCherry with Δ*icmT* mutant bacteria, the bacterial compartment was transiently labeled by the PtdIns(4)*P* probes (representing “baseline” PtdIns(4)*P* levels; see Fig. 1), but did not stably interact with the Golgi PtdIns(4)*P* pool (Fig. 6D). Taking the results together, the PtdIns(4)*P* probes 2×PH_FAPP_-GFP and P4C_SidC_-mCherry showed distinct and robust interaction dynamics with vacuoles harboring L. pneumophila JR32 (but not Δ*icmT* mutant bacteria), suggesting that LCVs accumulate Golgi-derived rather than plasma membrane-derived PtdIns(4)*P*.

### Transient Arf1 recruitment to LCVs.

**FIG 7:**
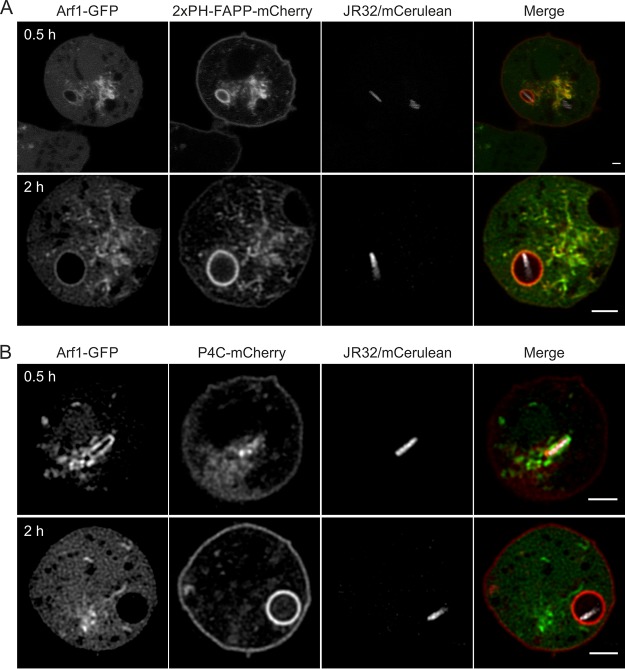
Transient Arf1 recruitment to LCVs. D. discoideum Ax3 amoebae producing Arf1-GFP (pWS036) and (A) 2×PH_FAPP_-mCherry (pWS035) or (B) P4C_SidC_-mCherry (pWS032) were infected (MOI 5, 0.5 h or 2 h) with L. pneumophila JR32 producing mCerulean (pNP099).

Arf1-GFP robustly localizes to LCVs at early time points of pathogen vacuole formation (30 to 60 min p.i.) (Fig. 5A). To further assess the time window during which Arf1 is recruited to LCVs, we infected D. discoideum strains producing Arf1-GFP and 2×PH_FAPP_-mCherry (Fig. 7A) or Arf1-GFP and P4C_SidC_-mCherry (Fig. 7B) with L. pneumophila JR32. These experiments confirmed Arf1 localization to LCVs at early time points; however, at 2 h p.i., the interaction of the Arf1-positive TGN with LCVs appeared to subside. This happened alongside the accumulation of the PtdIns(4)*P*/Golgi marker 2×PH_FAPP_, which remained on LCVs similarly to P4C_SidC_, likely reflecting the continuous accumulation of PtdIns(4)*P* on the LCVs. Hence, the interactions of LCVs with PtdIns(4)*P*-positive Golgi membranes occur early during infection (within 1 h p.i.) and later diminish. In summary, this high-resolution CLSM study using the Golgi markers Arf1, PH_FAPP_, and golvesin revealed that, during their maturation, LCVs interact with Golgi-derived PtdIns(4)*P*-positive vesicles at early time points of infection.

## DISCUSSION

Using real-time 3D high-resolution resonant CLSM, we have shown that vesicular trafficking contributes to the Icm/Dot-dependent removal and segregation of PtdIns(3)*P* as well as to the accumulation of PtdIns(4)*P* on LCVs. The PtdIns(3)*P*- and/or PtdIns(4)*P*-positive vesicles investigated here might correspond to the “smooth vesicles” associating with LCVs originally observed by EM (39). At early time points (<1 h p.i.) LCVs were not uniformly coated with a continuous PtdIns(4)*P* membrane, and the lumen of PtdIns(4)*P*-positive vesicles was still resolvable. The association of small PtdIns(4)*P*-positive vesicles with LCVs correlates with the punctate PtdIns(4)*P* and SidC staining observed previously (26, 28). The PtdIns(4)*P*-positive vesicles appeared to “stagnate” on the LCVs, thus leading to a net accumulation of the PI lipid. This process likely involves tethering and immobilization of PtdIns(4)*P*-positive vesicles on the LCVs, followed by fusion of the vesicle and the pathogen vacuole membrane. At present, the putative host and pathogen factors promoting the tethering of and interactions with PtdIns(4)*P*-positive vesicles are unknown.

The Golgi protein FAPP1 binds both PtdIns(4)*P* and Arf1 (24, 36, 40). Producing 2×PH_FAPP_-mCherry and Arf1-GFP or 2×PH_FAPP_-GFP and P4C_SidC_-mCherry, respectively, in D. discoideum indicated that the LCVs associate with the Golgi apparatus and accumulate Golgi-derived rather than plasma membrane-derived PtdIns(4)*P*. Most of the cellular PtdIns(4)*P* is found in the Golgi apparatus, the secretory vesicles, and the plasma membrane (22, 24), but there are additional pools of this lipid found in (late) endosomes (41, 42), which might contribute to the acquisition of vesicle-bound PtdIns(4)*P* by nascent LCVs. However, the fact that LCVs deviate from the endosomal route early during formation, together with the accumulation on LCVs of the Golgi-specific probes 2×PH_FAPP_-mCherry and Arf1-GFP, strongly suggests that the PtdIns(4)*P*-positive vesicles interacting with LCVs are indeed derived from the Golgi apparatus. Overall, these results also emphasize the importance of performing live-cell rather than fixed-sample experiments and strengthen the notion of the LCV as a dynamic compartment (co)defined by the frequency and/or duration of vesicular interactions. From a technological standpoint, the speed of the resonant scans and of multi-Z-plane imaging allowed us to decipher these processes.

At later stages of infection, the PtdIns(4)*P*-positive LCVs still interacted but did not fuse with PtdIns(3)*P*-positive vesicles. The Icm/Dot-translocated effector VipD shows Rab5-activated phospholipase A_1_ activity, removes PtdIns(3)*P* from endosomal membranes, and reduces Rab5 levels on early LCVs (43). Thus, VipD might contribute to limit the interactions of LCVs with endosomes throughout pathogen vacuole maturation. The putative Icm/Dot substrates promoting the observed early interactions of LCVs with PtdIns(4)*P*-positive vesicles and their sustained accumulation on the pathogen vacuole are unknown. In any case, the Icm/Dot substrate RalF is dispensable for the accumulation of PtdIns(4)*P* on LCVs. While Arf1-GFP was not observable on pathogen vacuoles harboring L. pneumophila Δ*ralF* (Fig. 5C), as published previously for mammalian cells (14), the accumulation of 2×PH_FAPP_-mCherry and, hence, PtdIns(4)*P* was not compromised. These results also indicate that Arf1, which recruits PI 4-kinase (see below), is dispensable for the accumulation of PtdIns(4)*P* on LCVs.

PI modulation during L. pneumophila infection and LCV formation is a complex process, likely involving the vesicle trafficking processes described here as well as L. pneumophila effectors. Several L. pneumophila Icm/Dot-translocated effectors have been described which might contribute to PI lipid metabolism directly on LCVs (27, 44). LepB, originally characterized as a Rab1 GTPase activating protein (GAP) (45–48), also exhibits PI 4-kinase activity and converts PtdIns(3)*P* to PtdIns(3,4)*P*_2_ (49). Furthermore, L. pneumophila produces the PI 3-phosphatases SidF (50), which preferentially hydrolyzes PtdIns(3,4)*P*_2_ and PtdIns(3,4,5)*P*_3_ *in vitro*, and SidP (51), which preferentially hydrolyzes PtdIns(3)*P* and PtdIns(3,5)*P*_2_ *in vitro*. LepB and SidF have been shown to contribute to the formation of PtdIns(4)*P* on LCVs in L. pneumophila-infected cells (49, 50), using the localization of the PtdIns(4)*P*-binding Icm/Dot substrate SidC as a readout (28). Interestingly, a novel family of translocated PtdIns 3-kinases which generate PtdIns(3)*P* from PtdIns (52) has recently been identified in *Francisella* (OpiA) as well as in L. pneumophila (LegA5). Finally, L. pneumophila produces an Icm/Dot-translocated phytase (inositol hexakisphosphate phosphatase), which produces PtdIns(4)*P* from the polyphosphorylated PI lipids PtdIns(3,4)*P*_2_, PtdIns(4,5)*P*_2_, and PtdIns(3,4,5)*P*_3_ *in vitro* (53). Although LppA appeared an ideal candidate to generate PtdIns(4)*P* on LCVs, no evidence was obtained to demonstrate that the phytase indeed modulates the pathogen vacuole PI pattern. In summary, a plausible sequence of events regarding the contribution of some L. pneumophila effectors to PI conversion on LCVs is as follows: The PI 3-kinase LegA5 and the PI 4-kinase LepB phosphorylate PtdIns and PtdIns(3)*P*, respectively, to produce PtdIns(3,4)*P*_2_, which is converted by the PI 3-phosphatase SidP to PtdIns(4)*P*.

Further adding to the complexity of the process, a number of host PI-metabolizing enzymes have been implicated in the production of PtdIns(4)*P* on the LCV membrane. The PI 4-kinase class IIIβ (PI4K IIIβ) is recruited by the small GTPase Arf1 and promotes traffic along the secretory pathway (54). Both Arf1 and PI4K IIIβ promote accumulation of SidC on the LCV, suggesting that these host factors contribute to PtdIns(4)*P* accumulation (30, 55). Arf1 localizes to LCVs (14), but the association of PI4K IIIβ with the pathogen vacuole remains to be assessed. Another host factor potentially involved in shaping the LCV PI pattern is the PI 5-phosphatase Oculocerebrorenal syndrome of Lowe (OCRL), which localizes to the TGN and endosomes and regulates retrograde trafficking between the two compartments (56). OCRL promotes intracellular replication of L. pneumophila (57) and determines LCV composition, including Rab1 and retrograde trafficking components (58). The PI 5-phosphatase preferentially dephosphorylates PtdIns(4,5)*P*_2_ and also PtdIns(3,4,5)*P*_3_, yielding PtdIns(4)*P* and PtdIns(3,4)*P*_2_. Based on the SidC localization assay, OCRL produces PtdIns(4)*P* on LCVs (57). Moreover, the PI 3-phosphatase effector SidF possibly cooperates with OCRL to produce PtdIns(4)*P* from PtdIns(3,4)*P*_2_.

Taken together, the available data are in agreement with a model stipulating that LCV PI conversion involves host factors as well as pathogen factors and is the sum of processes occurring in *trans* (at a distance from the LCV) and others occurring in *cis* (on the LCV directly). As documented in this study, vesicle identity and trafficking in *trans* seem to set the stage and determine early events of LCV formation. The L. pneumophila PI-modulating effectors appear to preferentially act in *cis*. Yet the issue of whether some of these effectors also act in *trans*, like several other L. pneumophila effectors, modifying, e.g., ribosomes, mitochondria, or histones (9, 10), has not been addressed. The work presented here provides an outline to address these issues and to search among the more than 250 uncharacterized L. pneumophila Icm/Dot substrates for effectors modulating early steps of LCV formation by interfering with host cell vesicle trafficking.

## MATERIALS AND METHODS

### Bacteria, cells, and growth conditions.

Bacterial strains and cell lines used are listed in Table 1. L. pneumophila strains were grown for 2 to 3 days on charcoal yeast extract (CYE) agar plates, buffered with *N*-(2-acetamido)-2-aminoethane sulfonic acid (ACES), at 37°C. Liquid cultures in ACES yeast extract (AYE) medium were inoculated at an optical density at 600 nm (OD_600_) of 0.1 and grown at 37°C for 16 to 21 h to the early stationary phase (2 × 10^9^ bacteria/ml). Chloramphenicol (Cam; 5 μg/ml) was added for plasmid retention.

**TABLE 1:** Strains and plasmids used in this study

Strain or plasmid	Relevant property(ies)^*a*^	Reference or source
D. discoideum Ax3	Parental strain	59
E. coli TOP10		Invitrogen
L. pneumophila GS3011	L. pneumophila JR32 *icmT3011*::Kan^r^ (Δ*icmT*)	60
L. pneumophila JR32	Virulent L. pneumophila serogroup 1 strain Philadelphia	61
L. pneumophila CR02	JR32 *ralF*::Kan^r^ (Δ*ralF*)	30

Plasmids		
pDM317	*Dictyostelium* extrachromosomal expression vector, N-terminal GFP, G418^r^	62
pDM323	*Dictyostelium* extrachromosomal expression vector, C-terminal GFP, G418^r^	62
pDM1044	*Dictyostelium* extrachromosomal expression vector, C-terminal mCherry, Hyg^r^	63
pDXA-HC	*Dictyostelium* expression vector, P*_act15_*, Neo^r^, Amp^r^	64
pGolvesin-GFP	Full-length gene encoding D. discoideum golvesin	37
pHKB95	pDM317-*gfp-2×FYVE*	H. Koliwer-Brandl et al., submitted for publication
pNP099	pMMB207-C, Δ*lacI*^q^ (constitutive *mCerulean*), Cam^r^	19
pNP102	pMMB207-C, Δ*lacI*^q^ (constitutive *mCherry*), Cam^r^	19
pPH_FAPP1	pEGFP-N1-*PH_FAPP1_-mCherry*	36 (gift of A. Helenius)
pRM010	pSW102-*PH_FAPP1_-gfp*, G418^r^	This work
pSE002	pDXA-*2×PH_FAPP1_-gfp*, G418^r^	This work
pSW102	pDXA-MCS-*gfp*, G418^r^	57
pWS032	pDM1044-*P4C_SidC_-mCherry*	19
pWS033	pDM323-*2×PH_FAPP1_-gfp*	This work
pWS034	pDM323-*P4C_SidC_-gfp*	58
pWS035	pDM1044-*2×PH_FAPP1_-mCherry*	This work
pWS036	pDM323-*Arf1*-*gfp*	This work
pWS038	pDM323-*Δ*(*1–75*;*119–579*)*golvesin-gfp*	This work

a Abbreviations: Amp, ampicillin; Cam, chloramphenicol; Hyg, hygromycin; Kan, kanamycin; G418, Geneticin.

D. discoideum Ax3 amoebae were cultivated in HL-5 medium (ForMedium) at 23°C in the dark. Cells were maintained every 2 to 3 days by rinsing once with fresh HL-5, washing off cells with 10 ml HL-5, and transferring 10% to 20% of the volume to a new T75 flask containing 10 ml medium. Cells were strictly maintained at between 30% and 90% confluence.

### Plasmid cloning.

All plasmids used are listed in Table 1. The pEGFP-N1-PH_FAPP1_-mCherry template was originally obtained from Ari Helenius and was cloned into pSW102 (pDXA-MCS-*gfp*), yielding pRM010. For plasmid pSE002 (pDXA-2×PH_FAPP_-GFP), the PH_FAPP_ gene was duplicated by two PCR amplifications using pRM010 as a template and the primer pairs oRM17 (5′-AAAAACGCGGTACCAAGGAGGGGGTGTTGTACAAGTGGAC-3′)/oSE003 (5′-AAAAACGCGGATCCTTGTCCTTGTGCTTTGGAGCTCCCCAGAGCGACCAGCCACC-3′) and oSE004 (5′-AAAAACGCGGATCCAAGGAGGGGGTGTTGTACAAGTGGAC-3′)/oSE002 (5′-AAAAACGCCTCGAGATGCTTTGGAGCTCCCCAGAGCGAC-3′). The fragments were cut with BamHI, ligated, digested with KpnI and XhoI, and inserted into pSW102 cut with the same enzymes. To construct plasmids pWS033 (2×PH_FAPP_-GFP) and pWS035 (2×PH_FAPP_-mCherry), the tandem PH_FAPP_ domain was amplified from pSE002 using primers oWS41 (5′-TCAGATCCCAAGCTAGATCTATGGATGGTACC-3′) and oWS42 (5′-CGCCCTTGCTCACCATACTAGTAGATGCTTTG-3′). The PCR fragment was cloned with BglII/SpeI into vectors pDM323 and pDM1044, respectively. To construct plasmid pWS036, Arf1 (ArfA) was PCR amplified from purified D. discoideum Ax3 cDNA (NBRP Nenkin, Tsukuba, Japan) using primers oWS53 (5′-TTTGGATCCATGGGTCTCGCTTTTGGTAAAC-3′) and oWS54 (5′-AAAACTAGTTTTTGAGGAGCTTGTTAAGGTATTTG-3′). The product was cloned with BamHI/SpeI into pDM323. To construct pWS038 [Δ(1–75;119–579)golvesin-GFP], the golvesin core fragment was amplified from template pGolvesin-GFP using primers oWS55 (5′-AAAAAGATCTATGTCAAATACAGGTAAAATATATTTAAG-3′) and oWS26 (5′-AAAAACTAGTATCAAATGGTAAACTAAAAACTAC-3′). The PCR fragment was cloned with BglII/SpeI into pDM323. All new vectors were transformed into Escherichia coli TOP10 for amplification and then sequenced.

### Transformation of Dictyostelium discoideum.

The D. discoideum parental strain Ax3 was grown to approximately 70% confluence. The HL-5 medium was discarded, and the flask was rinsed with 5 ml electroporation buffer (EB; 10 mM KH_2_PO_4_, 50 mM sucrose [pH 6.1], filter sterilized and stored at 4°C) without disturbing the cells. The rinse buffer was replaced with 5 ml fresh EB, and the cells were dislodged by the use of a 5-ml serological pipette. A 1-ml volume of cell suspension was added to each 4-mm-gap electroporation cuvette (Bio-Rad), and 4 to 5 µg of a given vector was mixed into the cuvette. For dually fluorescent strains, the two vectors were added to the cuvette simultaneously. Electroporation was performed with 2 pulses of 1 ms and 1 mV separated by a 5-s gap. Directly after electroporation, cells were transferred into a T75 flask containing 10 ml HL-5. At between 12 and 24 h after electroporation, the medium was replaced with fresh HL-5 and the required selection antibiotics were added. The medium was changed 72 h later. Upon the obvious appearance of several microcolonies (usually 6 to 7 days after transformation), cells were dislodged into fresh medium and transferred to a new flask.

### Sample preparation for microscopy.

D. discoideum amoebae producing the desired fluorescent probes were harvested from approximately 70%-confluent cultures. HL-5 medium was removed, and cultures were washed with 5 ml LoFlo medium (ForMedium) and resuspended in fresh LoFlo medium. The cells were seeded (300 μl) at a density of 2.5 × 10^5^/ml to 4 × 10^5^/ml in eight-well μ-slides (Ibidi). Cells were allowed to adhere for 1 h, after which the LoFlo medium was replaced. Infections (at a multiplicity of infection [MOI] of 5) with early stationary-phase cultures of L. pneumophila JR32 harboring pNP099 (mCerulean) or pNP102 (mCherry) were initiated in μ-slides already in position for imaging.

### Confocal laser scanning fluorescence microscopy setup.

All imaging was performed with living cells, carried out with a Leica TCS SP8 X CLSM with the following setup: white-light laser (WLL), 442-nm diode, HyD hybrid detectors for each channel used, HC PL APO CS2 63×/1.4 oil objective with Leica type F immersion oil, Leica LAS X software. mCerulean was excited at 442 nm and detected at around 469 nm. Enhanced GFP (EGFP) was excited at 487 nm and detected at around 516 nm. mCherry was excited at 587 nm and detected at around 622 nm. The microscope stage thermostat was set to hold the temperature at between 22°C and 25°C. Images were captured with a pinhole at between 0.6 and 0.9 Airy units (AU) and with a pixel/voxel size at or close to the instrument’s Nyquist criterion of approximately 39.5 × 39.5 × 118 nm (*xyz*).

Resonant scanning at 8,000 Hz (bidirectional scan) was used to capture videos corresponding to Fig. 1, 2, 3, 5, and 6B. Capture rates for 2 scans with 2 to 8 line averages were between approximately 2.5 and 5 frames per second. For Fig. 1, four Z-slices with 110-nm spacing were captured per time interval. Standard scanning at frequencies between 200 and 600 Hz (bidirectional scan with 2 to 3 line averages) was used to capture images and videos corresponding to Fig. 4, 6A, C, and D, and 7.

### Video and image processing.

All images were deconvolved with Huygens Professional version 17.10 (Scientific Volume Imaging, The Netherlands) using the CMLE algorithm with 40 iterations and a 0.05 quality threshold. Signal-to-noise ratios were estimated from the photons counted for a given image. Video captures and their snapshots were finalized with Imaris 9.1.0 software (Bitplane, Switzerland). Still images were finalized and exported with ImageJ software (https://imagej.nih.gov/ij/).
